# Magnetic Hybrid Nanosorbents for the Uptake of Paraquat from Water

**DOI:** 10.3390/nano7030068

**Published:** 2017-03-18

**Authors:** Tiago Fernandes, Sofia F. Soares, Tito Trindade, Ana L. Daniel-da-Silva

**Affiliations:** CICECO-Aveiro Institute of Materials, Department of Chemistry, University of Aveiro, 3810-193 Aveiro, Portugal; jtfernandes@ua.pt (T.F.); sofiafsoares@ua.pt (S.F.S.); tito@ua.pt (T.T.)

**Keywords:** paraquat, adsorption, bio-hybrids, magnetic nanoparticles

## Abstract

Although paraquat has been banned in European countries, this herbicide is still used all over the world, thanks to its low-cost, high-efficiency, and fast action. Because paraquat is highly toxic to humans and animals, there is interest in mitigating the consequences of its use, namely by implementing removal procedures capable of curbing its environmental and health risks. This research describes new magnetic nanosorbents composed of magnetite cores functionalized with bio-hybrid siliceous shells, that can be used to uptake paraquat from water using magnetically-assisted procedures. The biopolymers κ-carrageenan and starch were introduced into the siliceous shells, resulting in two hybrid materials, Fe_3_O_4_@SiO_2_/SiCRG and Fe_3_O_4_@SiO_2_/SiStarch, respectively, that exhibit a distinct surface chemistry. The Fe_3_O_4_@SiO_2_/SiCRG biosorbents displayed a superior paraquat removal performance, with a good fitting to the Langmuir and Toth isotherm models. The maximum adsorption capacity of paraquat for Fe_3_O_4_@SiO_2_/SiCRG biosorbents was 257 mg·g^−1^, which places this sorbent among the best systems for the removal of this herbicide from water. The interesting performance of the κ-carrageenan hybrid, along with its magnetic properties and good regeneration capacity, presents a very efficient way for the remediation of water contaminated with paraquat.

## 1. Introduction

Agriculture plays a critical role in the food supply and in the economy of any given country. With the overgrowth of the population, it becomes increasingly important to achieve a high-productive agriculture that can keep up with the demand. Up until the 1940s, most of the weed control was accomplished mechanically and with limited efficiency [1]. The advent of chemical herbicides during the 20th century had a great impact on controlling weeds that proliferate in agricultural and non-agricultural areas. Due to its high efficiency, rain fastness, and low price, paraquat has been a widely used herbicide since 1962, with a non-selective and fast action in killing green plant tissue upon contact. The great versatility of paraquat led to its overuse in several regions, placing non-targeted resources at risk of contamination by run-off or leaching. Although paraquat is generally inactivated by photodegradation and the non-reversible adsorption on clays or organic matter [2], several works have reported the presence of this herbicide in drinking-water sources [3,4,5,6]. The contamination of water can occur, for example, by the vertical transport of dissolved colloids (e.g., dispersed clays/organic matter) of accumulated paraquat through the soil [7,8]. In addition, its superior resistance to hydrolysis and its high water solubility significantly contribute to a greater risk of water contamination. The presence of paraquat in drinking water sources may represent a serious threat to human health, due to its acute toxicity level, which was the main reason that led to its ban in several countries [9]. In addition, this herbicide has been linked to several diseases in humans, including Parkinson’s disease [10,11,12].

It has become increasingly important to develop low-cost and highly efficient materials capable of removing this herbicide from water, because this is one of the main routes which exposes humans to paraquat. Most of the conventional removal methods are costly, less effective, and require specialized equipment or personnel (e.g., activated carbon, coagulation-flocculation, or membrane filtration) [13,14,15]. For this reason, several alternative methods have been explored for paraquat water remediation, including oxidation with Fenton’s reagent [16,17], photocatalysis [18], and adsorption [19,20,21]. Among these methods, adsorption has emerged with particular interest, thanks to its inherent simplicity and versatility [22,23,24]. Recently, materials such as three-dimensional graphene (3DG) [19], bimetallic Co-Fe nanoparticles [20], and carbon-coated polyacrylonitrile electrospun fiber membranes [21] have been investigated, for the removal of paraquat through adsorption. Despite its advantages, the information available in the literature regarding the application of bio-polymeric systems for the removal of paraquat from water, is scarce. Cocenza et al. described the preparation of alginate/chitosan membranes with the capability to remove paraquat from river water (*q*_e_: 10 mg·g^−1^) [25]. To the best of our knowledge, no other works have explored the capability of biopolymers to remove the herbicide paraquat from aqueous solutions.

**Figure 1:**
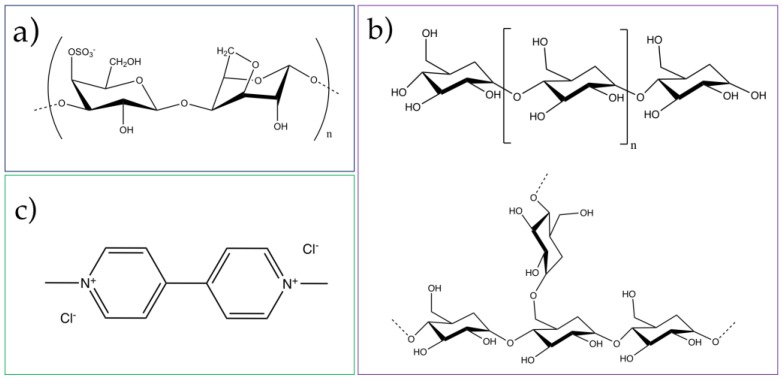
Structure of (**a**) κ-carrageenan; (**b**) starch, which contains (**top**) amylose (linear, 20 %); (**bottom**) amylopectin (branched, 80%) linked by glycosidic bonds and (**c**) paraquat.

The application of magnetic nanosorbents for the removal of herbicides remains a less explored area in the context of water purification. Our research group has reported a range of magnetic nanosorbents that are effective for the removal of distinct water pollutants, using magnetically-assisted procedures [26,27,28,29,30,31]. In particular, nanosorbents composed of magnetic cores and biopolymers at the surface are promising candidates for the removal of certain emerging organic pollutants from water, using magnetically-assisted technologies [32,33,34]. Therefore, in this work, the magnetic properties of iron oxide nanoparticles, coupled with the adsorption capacity of biopolymers, are exploited for the removal of paraquat from water. To this end, the biopolymers κ-carrageenan and starch (rice) were used to prepare magnetic hybrids (Figure 1). The adsorption performance of these nanomaterials was assessed by using distinct conditions, namely variable pH values, contact times, and herbicide concentrations.

## 2. Results and Discussion

### 2.1. Materials Synthesis and Characterization

The magnetic hybrid particles were prepared through the encapsulation of magnetic cores (about 50 nm diameter) with hybrid siliceous shells, containing the biopolymer covalently grafted onto the particle’s surface. Two distinct polysaccharides were investigated for surface modification, starch and κ-carrageenan. The magnetic cores were obtained by the alkaline oxidative hydrolysis of iron(II) sulfate, as previously reported [35]. The X-ray diffraction (XRD) patterns of the resulting iron oxide particles (Figure S1) agreed with the well-known diffraction patterns of magnetite (Fe_3_O_4_), indicating that this iron oxide was the predominant crystalline phase in the powdered samples [36]. In addition, the magnetic particles showed a magnetization hysteresis loop at room temperature, with saturation occurring at 84 emu/g [35]. The amorphous siliceous-biopolymer shell around the magnetite core was obtained through a one-step procedure, involving the hydrolysis and condensation of a mixture containing magnetic particles, the tetraethyl orthosilicate (TEOS), and an alkoxysilane with the biopolymer covalently bound via a urethane bond. As previously described, this synthetic approach allows for the preparation of spheroidal Fe_3_O_4_ particles, uniformly coated with siliceous hybrid shells that are extensively modified with κ-carrageenan molecules strongly grafted to the particle’s surfaces. Herein, we explore this strategy to obtain magnetite particles functionalized with starch [37]. For comparative purposes, particles without the biopolymer at the surface were also prepared in the absence of the silica-biopolymer precursor, by using TEOS as the SiO_2_ precursor.

**Figure 2:**
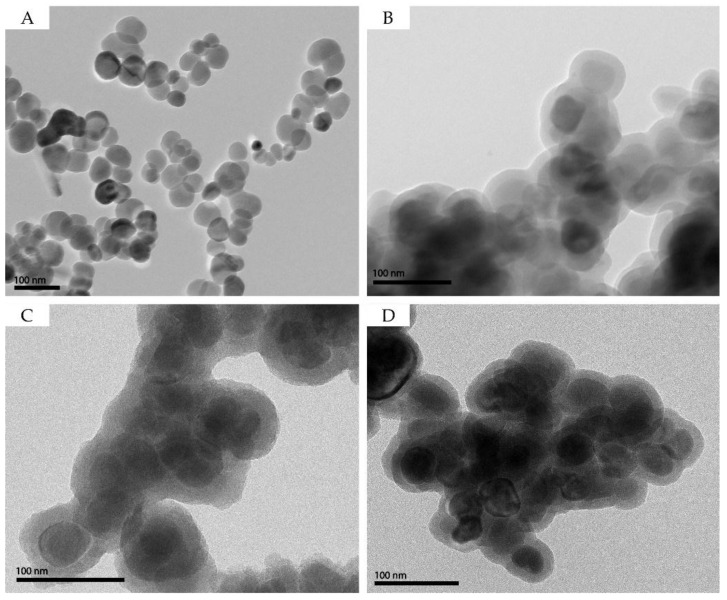
Transmission electron microscopy (TEM) images of (**A**) Fe_3_O_4_; (**B**) Fe_3_O_4_@SiO_2_; (**C**) Fe_3_O_4_@SiO_2_/SiStarch and (**D**) Fe_3_O_4_@SiO_2_/SiCRG.

A complete characterization of the particles was accomplished by assessing their morphological (Figure 2), chemical, and surface properties (Table 1). Transmission electron microscopy (TEM) images confirmed the spheroidal shape of magnetite cores with a nearly monodispersed size distribution (Figure 2A) and confirmed the presence of the siliceous shells after their surface coating (Figure 2B–D and Table 1). Accordingly, for the κ-carrageenan-, starch-, and silica-coated nanoparticles, the specific surface area decreased, due to an increase in the particle size after the formation of the shell. While the Fe_3_O_4_ and Fe_3_O_4_@SiO_2_ particles show a negligible carbon content, the particles containing the biopolymers exhibit >20 wt % of carbon. In addition, the Fe_3_O_4_@SiO_2_/SiCRG hybrid particles showed about a 4 wt % sulfur content, which can be assigned to the sulfonate groups of κ-carrageenan.

**Figure 3:**
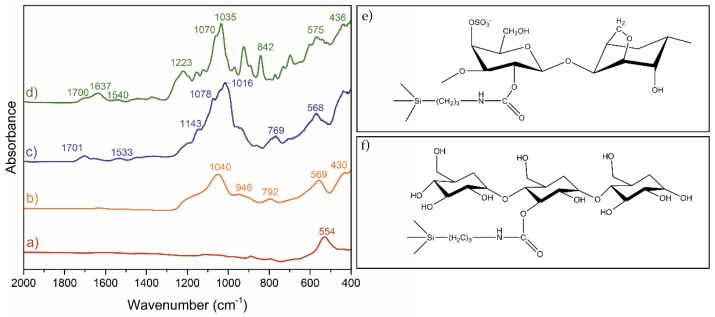
Attenuated total reflectance Fourier transform infrared spectroscopy (ATR-FTIR) spectra (400–2000 cm^−1^) of the samples: (**a**) Fe_3_O_4_; (**b**) Fe_3_O_4_@SiO_2_; (**c**) Fe_3_O_4_@SiO_2_/SiStarch; (**d**) Fe_3_O_4_@SiO_2_/SiCRG. Scheme illustrating the formation of urethane bonds in the biopolymer-silica matrix with (**e**) κ-carrageenan and (**f**) amylose from starch.

The chemical composition of the nanoparticles was further confirmed by attenuated total reflectance Fourier transform infrared spectroscopy (ATR-FTIR) (Figure 3, Table 2). The FTIR spectrum of the Fe_3_O_4_ cores shows a strong and large band at 554 cm^−1^, which can be assigned to the Fe–O lattice stretching vibration [38,39]. This band is also visible in the spectra of coated particles, although it is shifted to higher wavenumbers. The samples containing the biopolymers show the characteristic bands assigned to the organic counterpart, confirming their hybrid nature. Hence, for the starch containing sample, the FTIR spectrum shows bands at 1701 cm^−1^ (ν_C=O_) and 1533 cm^−1^ (ν_N–H_), confirming the formation of the covalent bond between starch and the siliceous network through urethane groups. In addition, the characteristic vibrational bands in the fingerprint region (800–1500 cm^−1^), along with several bands below 800 cm^−1^ (i.e., skeletal mode vibrations of glucose pyranose ring), further confirm the presence of starch in the particles [39]. For the Fe_3_O_4_@SiO_2_/SiCRG hybrids, several characteristic peaks of the biopolymer were also observed. The peaks at 930–1070 cm^−1^ (3,6-anhydro-d-galactose (DA)), 1220 cm^−1^ (ν_O=S=O_), and 840 cm^−1^ (d-galactose-4-sulphate (G4S)), are characteristic of κ-carrageenan [40,41,42,43]. In addition, the peaks at 436 cm^−1^ (δ_Si–O–Si_), 1540 cm^−1^ (δ_N–H_ and ν_C–N_), and 1700 cm^−1^ (H bonded ν_C=O_), confirm the formation of the siliceous network and the existence of urethane bonds, providing further evidence that the biopolymer is covalently bound to the network [44,45,46,47]. As previously reported, the band at 1637 cm^−1^ in κ-carrageenan hybrid particles can arise from ν_C=O_ of urea, formed during synthesis due to residual moisture in the polysaccharide, suggesting the coexistence of urea and urethane bonds in the hybrid particles [37]. For simplicity, all of the most relevant peaks are summarized in Table 2.

### 2.2. Uptake of Paraquat from Water

#### 2.2.1. Effect of Shell Chemical Composition

**Figure 4:**
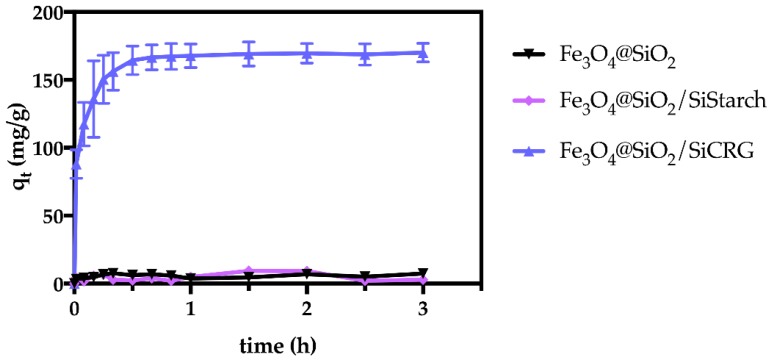
The performance of the bio-hybrids for the removal of paraquat (90 μg/mL) from water at pH 7.3, 25 °C.

The performance of the hybrid particles was investigated for the same herbicide concentration (90 μg/mL) at pH 7.3, with 3 h contact time. Based on the results presented in Figure 4, the hybrids containing κ-carrageenan exhibited a superior adsorption capacity when compared with those coated with starch. To understand the role of the biopolymers in paraquat removal, particles exclusively containing the silica shell were also investigated under the same conditions. Since the particles without biopolymer (Fe_3_O_4_@SiO_2_) also exhibit a low removal capacity, it is assumed that the good performance of the κ-carrageenan hybrids arises thanks to the presence of the biopolymer at the surface. Control experiments (i.e., without particles) were also carried out in parallel and revealed a negligible loss of herbicide during all the experiments (Figure S4). Thus, the observed removal capacity was exclusively attributed to the Fe_3_O_4_@SiO_2_/SiCRG hybrid particles through adsorption. Figure 4 shows that, after 3 h, the paraquat uptake was 95% using Fe_3_O_4_@SiO_2_/SiCRG sorbents and much less, <5%, with Fe_3_O_4_@SiO_2_/SiStarch particles. A possible explanation for the good performance of Fe_3_O_4_@SiO_2_/SiCRG particles is that the κ-carrageenan at the surface provides anionic surface groups (sulfonate groups, see Figure 1) that can interact with the herbicide paraquat, whose molecules at pH 7.3 are in the cationic form. Moreover, since paraquat is a bypiridilium dication with high electron affinity, it acts as a Lewis acid. Thus, paraquat is readily available to interact with electron rich species, such as anions or organic groups with lone pairs [48,49]. Indeed, it has been previously reported that paraquat strongly interacts with polymers containing anionic groups [49,50], mainly by electrostatic interaction. Conversely, despite the negative zeta potential of starch hybrids (ξ = −45.4 mV at pH 7.3, see Figure S4), their inferior performance may be explained by the lower electron density of their exposed hydroxyl groups. This reduced availability of electron lone pairs impacts their capacity to adsorb paraquat (see Figure 1).

#### 2.2.2. Effect of Contact Time and Paraquat Concentration

**Figure 5:**
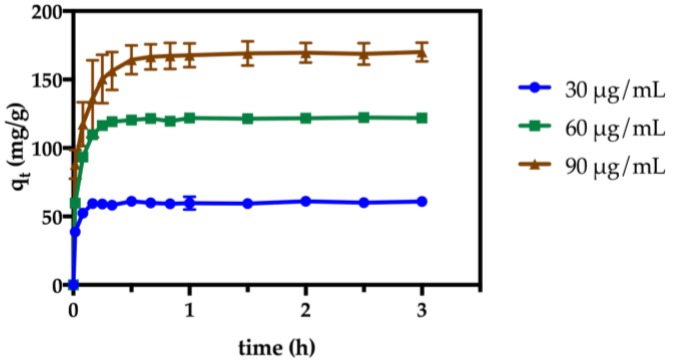
Time profile of adsorption capacity of κ-carrageenan hybrids at variable paraquat initial concentrations (pH 7.3, 25 °C).

Our subsequent studies have been accomplished for the κ-carrageenan containing nanosorbents, due to their superior performance as nanosorbents for paraquat. Consequently, the effect of herbicide concentration was assessed in the concentration range of 30–90 μg/mL at pH 7.3 (Figure 5). It was found that 3 h of contact time was enough to achieve the optimal performance, after which the adsorption capacity reached its maximum (i.e., 95% of removal, Figure S5). The results presented in Figure 5 confirm that, despite the herbicide concentration, the adsorption profile remains unchanged. In general terms, the adsorption is characterized by fast kinetics, where the maximum performance is achieved after 30 min. Similar adsorption profiles have been reported for paraquat removal using other types of sorbents [19,20].

#### 2.2.3. Kinetic Modelling

**Figure 6:**
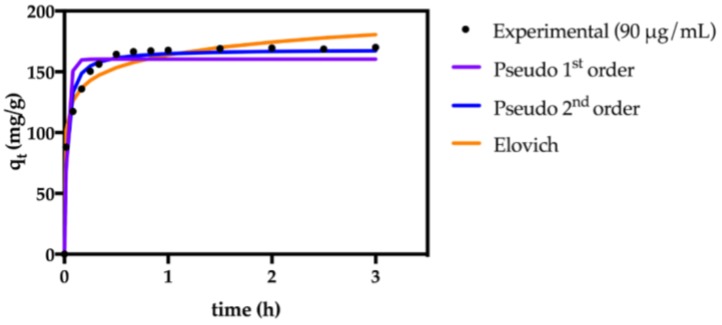
Modeling of adsorption kinetics of paraquat onto Fe_3_O_4_@SiO_2_/SiCRG particles using pseudo 1st and 2nd order kinetic equations and the Elovich kinetic model, for an initial paraquat concentration of 90 μg/mL.

The kinetic adsorption data were fitted to three distinct kinetic equations commonly used in the study of adsorption processes: the pseudo-first order equation [51], the pseudo-second order equation [52], and the Elovich kinetic models [53] (see Supporting Information for model equation). The kinetic parameters and the evaluation of the goodness of the fits, obtained by non-linear regression analysis, are reported in Table 3, and the kinetic fittings are shown in Figure 6 and Figure S4 (Supporting Information).

The pseudo-second order fitting (Equation (S6)) was the one with the highest correlation coefficient (*R*^2^), indicating a good agreement with the experimental data. The goodness of this fit is further confirmed by the values of χ^2^, being the lowest for the pseudo-2nd order model. These results suggest that the adsorption of paraquat onto the κ-carrageenan particles follows a pseudo-second order kinetics model, meaning that the chemisorption is the rate-limiting step [54]. Thus, the adsorption rate is most likely controlled by the electrostatic interaction between the anionic sulfonate groups of κ-carrageenan and the cationic paraquat molecules. Similar observations have been reported regarding this type of kinetic profile of paraquat adsorption for other materials containing surface anionic groups [19,21].

#### 2.2.4. Effect of pH on Adsorption in Aqueous Medium

**Figure 7:**
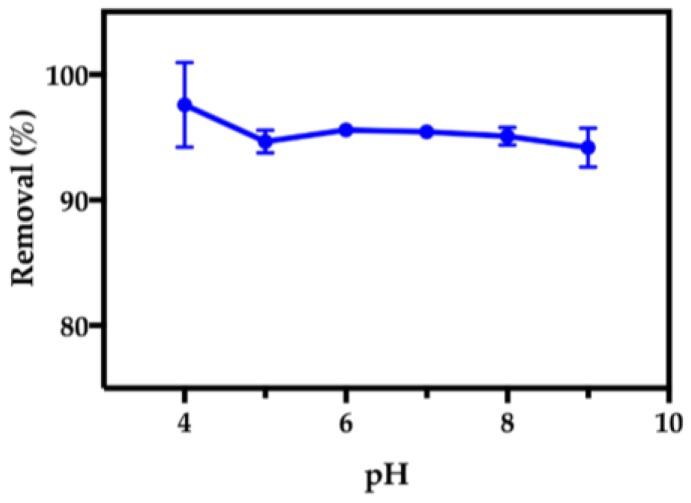
The effect of pH on the removal of paraquat from water using the Fe_3_O_4_@SiO_2_/SiCRG hybrids.

The performance of κ-carrageenan hybrid particles was investigated in the pH range 4–9 for 24 h contact time (Figure 7). The optimal pH range for the removal of paraquat was within 6–8, by also taking into consideration the realistic working pH conditions, the isotherm measurements were carried out at pH 7.3. In addition, previous studies have shown that the Fe_3_O_4_@SiO_2_/SiCRG hybrids exhibit a good chemical stability at pH 7, possessing a negligible leaching of Fe ions from the magnetic core [33]. At this pH range, the Fe_3_O_4_@SiO_2_/SiCRG hybrids possess a negative surface charge (ξ = −44.6 mV) (Figure S3), due to the presence of ionized sulfonate groups at the surface of the nanoparticles [37]. On the other hand, paraquat appears as bipyridinium molecular cations, with two permanent positive charges in the whole pH range [55]. Considering the chemical nature of the hybrids and the herbicide, it is expected that the intermolecular interactions between them is most likely to occur via electrostatic interactions involving the permanent charges of the biopolymer (sulfonate groups) and the bipyridinium cations [49,56].

#### 2.2.5. Isotherm Studies and Thermodynamic Parameters

**Figure 8:**
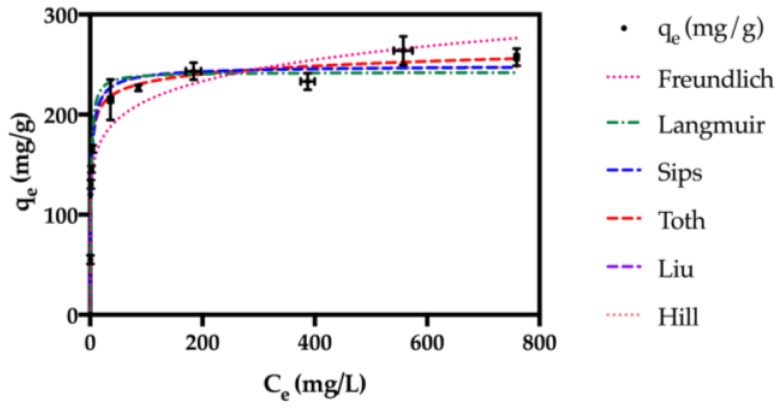
Isotherm data and corresponding model fitting for the adsorption of paraquat on the Fe_3_O_4_@SiO_2_/SiCRG biosorbents.

Isotherm studies were accomplished for the Fe_3_O_4_@SiO_2_/SiCRG hybrids and the data plotted (Figure 8), based on the equilibrium adsorption amount of paraquat (*q*_e_) as a function of the equilibrium concentration of this herbicide (*C*_e_) [22,57]. The data were fitted to Langmuir [58] and Freundlich [59] isotherms, which are two-parameter isotherms, and to Sips [60], Toth [61], Liu [62], and Hill [63] isotherms, which are three-parameter isotherms (see Supporting Information for model equation).The data were fitted to several isotherm equations in the non-linear form, using GraphPad Prism version 7.00. The goodness of the fit was determined, based on the correlation coefficient (*R*^2^) and the Chi-square test value (χ^2^). It has been shown before that the Chi-square analysis provides a better indication of the fit for non-linear regression analysis [64]. The model parameters and goodness of the fit are depicted in Table 4.

The *R*^2^ and χ^2^ values indicate that the two-parameter model that best fits the experimental data is the Langmuir isotherm. The monolayer adsorption capacity predicted by this model was 242.4 mg·g^−1^. The adsorption process is most likely to occur by a monolayer, instead of the multilayer adopted in the Freundlich model, because better fitting parameters were found by applying the Langmuir isotherm.

Based on the fitting indicators, the Toth isotherm is the three-parameter model that best describes the experimental isotherm data. This indicates that the nanosorbent is characterized by heterogeneous adsorption sites, where most of the sites possess an adsorption energy lower than the maximum or mean adsorption energy (i.e., asymmetrical quasi-gaussian energy distribution with strong tail for low adsorption energies) [22,61].

To understand the nature of the interaction between paraquat and the Fe_3_O_4_@SiO_2_/SiCRG biosorbents, the standard free energy change (∆*G*°) was determined from the isotherm experimental data obtained at 25 ± 1.0 °C (see Supporting Information) [65,66]. The calculated ∆*G*° was −25.2 kJ/mol, indicating a spontaneous adsorption of paraquat on the surface of the biosorbents. A similar profile has been attributed to other types of materials, where a spontaneous adsorption of paraquat was also observed [67,68].

#### 2.2.6. Desorption and Reuse

**Figure 9:**
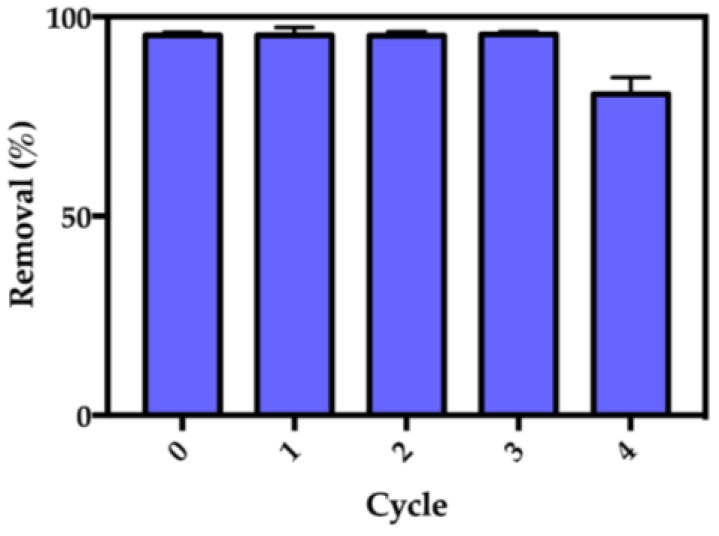
The reuse performance of the Fe_3_O_4_@SiO_2_/SiCRG hybrids in the removal of paraquat from water.

The regeneration capacity of the Fe_3_O_4_@SiO_2_/SiCRG hybrids was assessed by running four adsorption cycles (Figure 9). The use of KCl (1 M) provided a way of regenerating the particles in mild conditions and within a few minutes. This is a simple cationic exchange treatment, based on the high affinity of the sulfonate groups to potassium ions [69]. Hence, the paraquat-loaded nanosorbents were washed four times with KCl aqueous solution, where most of the herbicide was successfully desorbed in the first two rising steps. No paraquat was detected after the last step and after rinsing with deionized water.

The results presented in Figure 9 indicate that the Fe_3_O_4_@SiO_2_/SiCRG hybrids possess a great reuse capacity, where a decrease in the performance was only observed at the 4th cycle (i.e., from 95% to 78%). This decrease in the adsorption capacity may be attributed to any structural changes in the biopolymer caused by the K^+^ ions, which can reduce the availability of sulfonate groups to interact with the herbicide. The loss of biopolymer during the rinsing steps is less likely to occur since κ-carrageenan was covalently linked to the surface of the particles.

#### 2.2.7. Comparison with Other Sorbents

The Fe_3_O_4_@SiO_2_/SiCRG hybrids exhibit a maximum adsorption capacity (*q*_max_) of 257 mg·g^−1^ (experimental value). As shown in Table 5, several works have also reported the use of other materials for the removal of paraquat from water. The magnetic carrageenan silica hybrids reported in this work exhibit a high efficiency, with the ability for regeneration (four cycles). The materials are among the best (in fact second) sorbents for this pesticide and show the great advantage of magnetic separation.

## 3. Materials and Methods

### 3.1. Chemicals

Ferrous sulfate heptahydrate (FeSO_4_·7H_2_O) (>99%) and ethanol (CH_3_CH_2_OH) (>99%) were obtained from Panreac (Barcelona, Spain). Paraquat dichloride (C_12_H_14_Cl_2_N_2_, >98%), starch (from rice), Tetraethyl orthosilicate (Si(OC_2_H_5_)_4_, TEOS, >99%), potassium nitrate (KNO_3_) (>99%), and 3-(triethoxysilyl)propyl isocyanate ((C_2_H_5_O)_3_Si(CH_2_)_3_NCO, ICPTES, 95%) were purchased from Sigma-Aldrich (Steinheim, Germany). Milli-Q water was obtained from the Synergy equipment from Millipore with a 0.22 μm filter (Darmstadt, Germany). Ammonia solution (25% NH_3_) was purchased from Riedel-de-Häen (Hanover, Germany) and potassium hydroxide (KOH) (>86%) was purchased from Pronolab (Lisbon, Portugal). *N*,*N*-Dimethylformamide (HCON(CH_3_)_2_) and ascorbic acid (>99%) were obtained from Carlo Erba Reagents (Peypin, France). Methanol (CH_3_OH) (>99%) was purchased from VWR International (Fontenay-sous-Bois, France) and κ-Carrageenan (300,000 g/mol) was obtained from Fluka Chemie (Steinheim, Germany). Unless clearly stated, all chemicals were used without any further treatment.

### 3.2. Synthesis of Hybrids

All materials were prepared following the conditions previously reported by our group [33,37]. In general terms, all hybrid particles were synthesized in a two-step approach. Initially, the magnetite cores with an average size of 50 nm (Figure S2) were prepared by alkaline oxidative hydrolysis of an iron(II) salt (FeSO_4_·7H_2_O) [35]. In a second step, the magnetic particles were encapsulated with the biopolymer-silica hybrid shell through alkaline catalyzed hydrolysis and the condensation of a mixture of TEOS and the precursor, which is an alkoxysilane with the biopolymer covalently linked (SiBP) [33]. These SiBP precursors were prepared by reacting the corresponding biopolymers (i.e., κ-carrageenan and starch (rice)) with the silane coupling agent ICPTES [37].

### 3.3. Quantification of Paraquat

Although paraquat absorbs in the UV region (256 nm), its direct quantification is usually unreliable due to the intense absorption, even at very low concentrations. Thus, the quantification of paraquat through UV/Vis is usually accomplished by a derivatization step.

In this work, the derivatization of paraquat was investigated by the reduction of the nitrogen atoms in the aromatic ring, giving rise to a blue radical that absorbs at 600 nm. To this end, three reducing agents (ascorbic acid, glucose, and sodium borohydride) were explored, based on the conditions already reported by Gupta et al. [74,75,76]. Since the reduced paraquat is unstable and the reaction can be reversed by the presence of oxygen [77,78], the quantification of paraquat was accomplished by the in-situ reaction in the UV/Vis cuvette, which was tightly closed. The best calibration curve (highest *R*^2^ values) was obtained when reducing paraquat with ascorbic acid (AA) and potassium iodate (KIO_3_) in a molar ratio of 3:1. The KIO_3_ was added to reduce the ascorbic acid into dehydroascorbic acid and provide a better stability to the reducing mixture [78,79].

Briefly, to a fraction of solution containing paraquat, 340 μL of 0.5 wt % aqueous solution of AA:KIO_3_ and 680 μL of 0.3 M NaOH, were added. The reaction was left to develop at 35 ± 0.1 °C for 3 minutes and the absorbance was recorded at 600 nm. A calibration curve was obtained based on this method, with linearity in the concentration range of 0.625 to 10 μg/mL (Figure S8). All the aqueous solutions were prepared in ultra-pure water.

### 3.4. Removal of Paraquat from Water

The performance of the hybrid nanosorbents was investigated by several adsorption experiments. All the experiments described in the next sections were carried out in glass vials and accomplished in duplicate. The aqueous solutions of the herbicide were freshly prepared before each experiment in ultra-pure water. When required, the pH of the solutions was adjusted using NaOH (0.01 M) and HCl (0.01 M).

For all the experiments, the corresponding volumes of herbicide solutions at a known concentration were added to defined quantities of hybrid nanosorbents (ratio of 2:1, Solution:NPs). The vials were then shaken using a vertical rotator at a constant speed (30 rpm) and temperature (25 ± 2 °C). The starting point was considered when the vials with the herbicide/hybrids were exposed to these conditions (*t*_0_).

The quantity of herbicide adsorbed, i.e. the adsorption capacity (*q_t_*, mg·g^−1^) and the removal capacity (*R*, %) of the hybrids were calculated using Equations (1) and (2), respectively:

(1) $$q_{t}=\left(C_{0}-C_{t}\right)\times \frac{V}{m},$$

(2) $$R=\left(C_{0}-C_{t}\right)/C_{0}\times 100,$$

where, *C*_0_ is the initial concentration (μg/mL), *C_t_* is the concentration of the herbicide at time *t* (μg/mL), *V* is the total volume of herbicide solution (mL), and m is the mass of hybrid nanosorbents (mg).

Control experiments (i.e., without hybrids) were also carried out to confirm that the loss of herbicide through degradation or glass adsorption was negligible in the investigated time intervals. The paraquat of all aliquots taken during the removal experiments was derivatized according to the procedure described in the previous section and quantified through UV/Vis.

#### 3.4.1. Effect of pH

To verify the best pH values where the highest removal performance is achieved, herbicide solutions (30 µg/mL) within the pH range of 4 to 9 (5 mL) were prepared and put in contact with the hybrids (2.5 mg) for 24 h, at the conditions described in the previous section. After that time, an aliquot of the solution was taken and quantified following the procedure described in Section 3.3.

#### 3.4.2. Effect of Contact Time

Overall, 40 mL of paraquat solution, at a pre-defined concentration and pH 7, was added to 20 mg of hybrid nanosorbents. This mixture was then transferred to the conditions referred to in the Section 3.4. Several aliquots were taken along the time where the adsorption capacity of the hybrids (*q_t_*) was plotted against contact time.

#### 3.4.3. Equilibrium Isotherms Experiments

The equilibrium isotherms for the removal of paraquat were obtained by dispersing ca. 2.5 mg of nanosorbent particles in 5 mL solutions containing different concentrations of herbicide (30, 60, 70, 80, 90, 150, 200, 300, 700, and 900 µg/mL) at pH 7.3. The experiments were conducted for one hour and the amount of remaining herbicide in solution was determined through UV/Vis.

#### 3.4.4. Regeneration Experiments

For the regeneration experiments, the Fe_3_O_4_@SiO_2_/SiCRG hybrids were put in contact with the herbicide at 90 μg/mL (pH 7.3, 25 °C), for five cycles. During each cycle, the herbicide was desorbed by washing the hybrids several times with KCl (1 M), deionized water, and ethanol. The successfulness of the desorption was always confirmed by assessing the presence of paraquat in solution through UV/Vis during the washing steps.

### 3.5. Instrumentation

The elemental analysis of carbon, nitrogen, hydrogen, and sulfur was obtained on a Leco Truspec-Micro CHNS 630-200-200. The specific surface area of the particles was assessed by nitrogen adsorption Brunauer–Emmett–Teller (BET) measurements, performed with a Gemini V2.0 Micromeritics instrument (Norcross, Georgia, USA). The pore size was evaluated from the desorption branch using the Barret–Joyner–Halenda (BJH) method and the pore volume was calculated from the adsorbed amount. The Fourier transform infrared (FTIR) spectra of the particles were measured in the solid state. The spectra of the materials were collected using a Bruker Optics Tensor 27 spectrometer (Billerica, MA, USA) coupled to a horizontal attenuated total reflectance (ATR) cell, using 256 scans at a resolution of 4 cm^−1^. The morphology and size of the particles were analyzed by transmission electron microscopy (TEM), using a Hitachi H-9000 TEM microscope operating at 300 kV (Chiyoda, Tokyo, Japan). Samples for TEM analysis were prepared by evaporating the diluted suspensions of the nanoparticles on a copper grid coated with an amorphous carbon film. The surface charge of the nanoparticles was assessed by zeta potential measurements performed in aqueous solutions of the particles, using Zetasizer Nano ZS equipment from Malvern Instruments (Malvern, UK). A Perkin Elmer Analyst 100 apparatus was employed for the iron quantification. The paraquat uptake experiments were performed using a Grant Bio PTR-25 360 Vertical Mini Rotator (Cambridge, UK). The paraquat concentration was determined spectrophotometrically, using a Jasco U-560 UV/Vis spectrophotometer (Easton, MD, USA) and ultrapure water as the reference.

## 4. Conclusions

Two types of magnetic nanosorbents with distinct surface chemistries have been reported here. These nanosorbents comprise magnetite nanoparticles coated with hybrid siliceous shells and a biopolymer: κ-carrageenan or starch. The ability of these nanosorbents to remove the herbicide paraquat from water was investigated under several operational conditions. In the conditions investigated here, the κ-carrageenan (Fe_3_O_4_@SiO_2_/SiCRG) containing nanosorbents have shown a superior adsorption capacity towards paraquat, when compared to the starch analogues. The equilibrium adsorption data of these nanoparticles show good agreement to the Langmuir and Toth isotherms, resulting in an experimental maximum adsorption capacity of 257 mg·g^−1^. It is worth noting that the Fe_3_O_4_@SiO_2_/SiCRG materials reported here are among the best sorbents reported for paraquat and show an ability for regeneration. Therefore, these nanosorbents offer new possibilities for the purification of water contaminated with paraquat, namely by applying magnetic-assisted cleaning technologies.

## Figures and Tables

**Table 1:** Physicochemical properties of magnetic nanohybrids and magnetic particles without biopolymer (*D* = diameter; *T* = thickness; *S*_BET_ = Specific surface area; *V*_p_ = Pore volume).

Sample	*D*: Core (nm)	*T*: Shell (nm)	C (%)	H (%)	N (%)	S (%)	*S*_BET_ (m^2^·g^−1^)	*V*_p_ (cm^3^·g^−1^)
Fe_3_O_4_	54 ± 8	-	0.08	0.34	0.17	-	52.7	-
Fe_3_O_4_@SiO_2_	54 ± 8	13 ± 2	0.23	0.37	0.01	-	35.8	0.045
Fe_3_O_4_@SiO_2_/SiStarch	54 ± 8	19 ± 4	27.43	4.68	1.01	-	42.1	0.022
Fe_3_O_4_@SiO_2_/SiCRG	54 ± 8	14 ± 2	20.39	3.69	0.48	4.32	38.4	0.043

**Table 2:** Selected vibrational bands of the materials investigated in this work.

Assignment	Fe_3_O_4_	Fe_3_O_4_@SiO_2_	Fe_3_O_4_@SiO_2_/SiStarch	Fe_3_O_4_@SiO_2_/SiCRG
δ(Si–O–Si)	-	430 (s)	-	436 (s)
ν(Fe–O)	554 (vs)	569 (vs)	568 (vs)	575 (vs)
Pyranose ring	-	-	500–800 (m)	-
ν_s_(O–Si–O)	-	792 (vw)	-	-
G4S	-	-	-	842 (s)
νSi–OH	-	946 (vw)	-	-
ν_a_(Si–O–Si)	-	1040 (vs)	-	-
DA (C–H and C–OH)	-	-	-	930–1070 (vs)
ν_a_(O=S=O)	-	-	-	1223 (s)
δ(N–H), ν(C–N)	-	-	1533 (vw)	1540 (vw)
ν(C=O) urea	-	-	-	1637 (m)
ν(C=O) urethane	-	-	1701 (w)	1700 (w)

* vs: very strong; s: strong; m: medium; w: weak; vw: very weak; ν: stretching vibration; ν_a_: asymmetric stretching vibration; δ: deformation vibration.

**Table 3:** Kinetic parameters estimated from pseudo 1st order, pseudo 2nd order, and Elovich models and evaluation of the respective fittings for initial paraquat concentrations (*C*_0_): 30, 60 and 90 μg/mL.

*C*_0_ (μg/mL)	Pseudo 1st Order *			Pseudo 2nd Order **			Elovich Model ***		
	*R*^2^ (χ^2^)	*k*_1_	*q*_e_	*R*^2^ (χ^2^)	*k*_2_	*q*_e_	*R*^2^ (χ^2^)	α	β
30	0.9842 (0.9490)	1.04	59.29	0.9960 (0.2489)	0.03	60.76	0.9406 (7.0355)	20236.6	0.20
60	0.9666 (6.5473)	0.52	118.97	0.9949 (0.8727)	0.01	123.81	0.9417 (9.3153)	20236.6	0.10
90	0.9171 (19.9320)	0.56	160.35	0.98870 (2.2631)	0.00	168.53	0.9703 (4.1783)	12117.3	0.07

* *k*_1_ (min^−1^); *q*_e_ (mg·g^−1^); ** *k*_2_ (g·mg^−1^·min^−1^); *q*_e_ (mg·g^−1^); *** α (mg·g^−1^·min^−1^) β (g·mg^−1^).

**Table 4:** Isotherm models that were fit for the adsorption of paraquat using the κ-carrageenan nanoparticles as the nanosorbent.

Isotherm	Model Parameters			Goodness of fit	
**Langmuir**	*q*_L_ (mg·g^−1^)	*K*_L_ (L·mg^−1^)		*R*^2^	χ^2^
	242.4	0.6615		0.9645	16.65
**Freundlich**	*K*_F_ (mg^(1−1/*n*)^·L^(1/*n*)^·g^−1^)	1/*n*		*R*^2^	χ^2^
	119.0	0.1271		0.9170	25.46
**Sips**	*q*_max_ (mg·g^−1^)	*K*_S_ (mg·L^−1^)^−1/*n*^*_S_*	*n*_S_	*R*^2^	χ^2^
	170.6	0.6827	0.7449	0.9689	15.90
**Toth**	*q*_max_ (mg·g^−1^)	*K*_T_ (mg·L^−1^)*^n^_T_*	*n_T_*	*R*^2^	χ^2^
	200.6	0.8779	0.9562	0.9745	14.38
**Liu**	*q*_max_ (mg·g^−1^)	*K*_g_ (L·mg^−1^)	*n*_L_	*R*^2^	χ^2^
	249.8	0.5990	0.7449	0.9689	15.90
**Hill**	*q*_max_ (mg·g^−1^)	*K*_H_ ((mg·L^−1^)*^n^*_H_)	*n*_H_	*R*^2^	χ^2^
	249.8	1.4650	0.7449	0.9689	15.90

**Table 5:** Comparison of *q*_max_ values for Fe_3_O_4_@SiO_2_/SiCRG and other reported adsorbents for paraquat removal from water. All *q*_max_ values are presented, based on experimental data.

Sorbent	*q*_max_ (mg·g^−1^)	*T* °C	pH	Reference
3D Graphene	119/604	24	6/12	[19]
Co-Fe Microgel	190 *	RT (n.a.)	n.a.	[20]
Carbon fiber membranes	437.64	20	7	[21]
Sawdust	9.47	25	n.a.	[67]
Algerian Clays (bentonite)	100	25	n.a.	[70]
MAA Rice Husk	200	37.5	n.a.	[71]
Rice Husk Silica	185.2	n.a.	n.a.	[72]
Activated Carbon	7.52	25	7	[73]
Fe_3_O_4_@SiO_2_/SiCRG	257	25	7.3	Our work

* Value obtained from kinetic studies.

## Supplementary Materials

The following are available online at http://www.mdpi.com/2079-4991/7/3/68/s1.
