# Supplementary material for: Magnetic Hybrid Nanosorbents for the Uptake of Paraquat from Water
Source: Nanomaterials (Basel). 2017 Mar 18;7(3):68. doi: 10.3390/nano7030068 (PMC5388170; doi:10.3390/nano7030068)
Supplement: Supplementary file 1 [file nanomaterials-07-00068-s001.pdf]

Supplementary Materials: Magnetic Hybrid Nanosorbents for the Uptake of Paraquat from Water

Tiago Fernandes, Sofia F. Soares, Tito Trindade and Ana L. Daniel-da-Silva

A. Materials Characterization

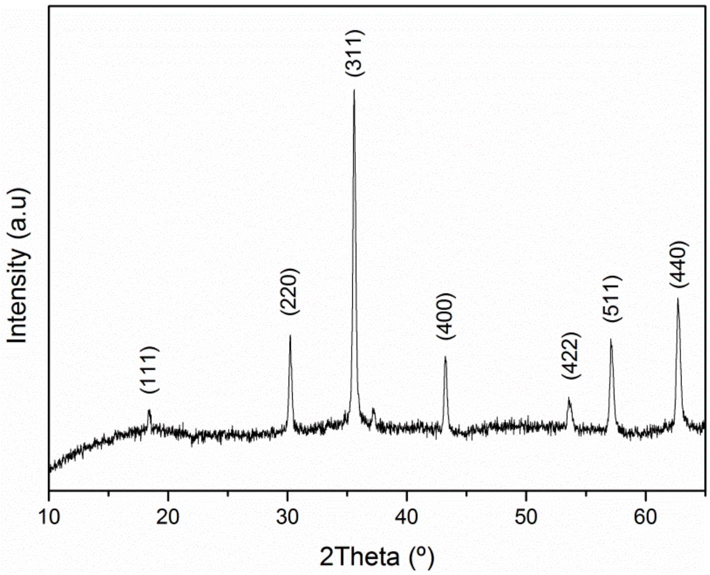

Figure S1. XRD pattern of magnetite (Fe_3O_4) nanoparticles.

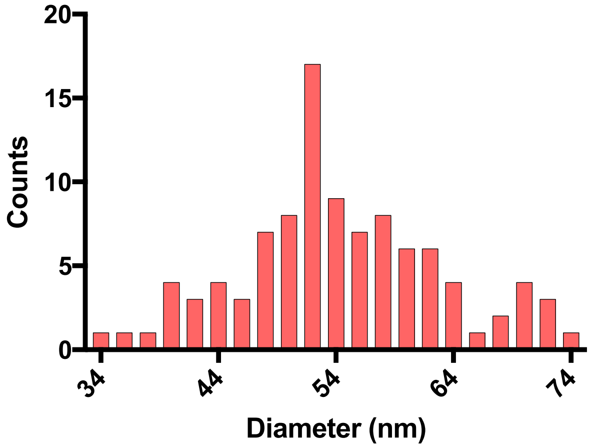

Figure S2. Particle size distribution for the Fe_3O_4 nanoparticles, assessed by TEM measurements.

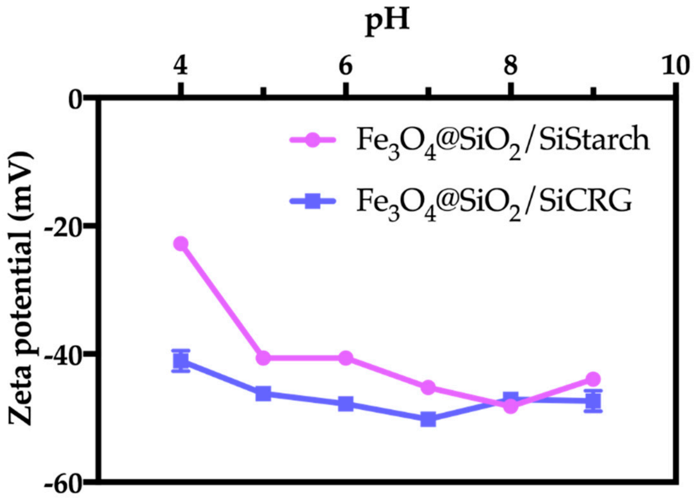

Figure S3. Zeta potential of $Fe_3O_4@SiO_2$/SiStarch and $Fe_3O_4@SiO_2$/SiCRG nanoparticles in the pH range 4-9.

B. Paraquat removal experiments

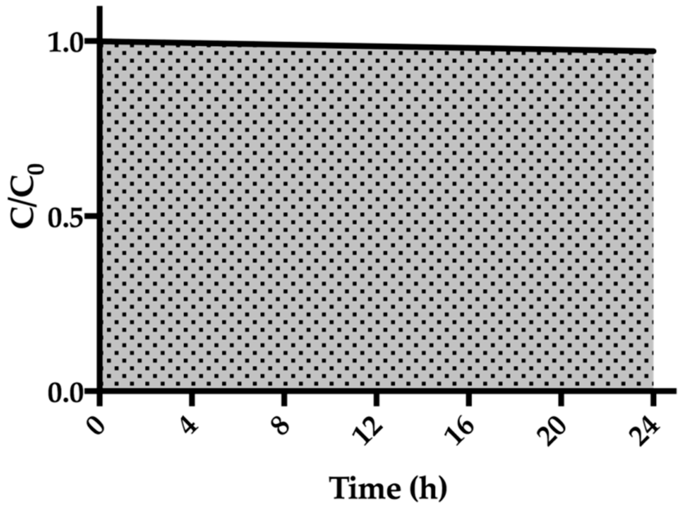

Figure S4. Control experiments carried out for 24 hours, revealed a negligible loss of paraquat either by glass adsorption or hydrolysis.

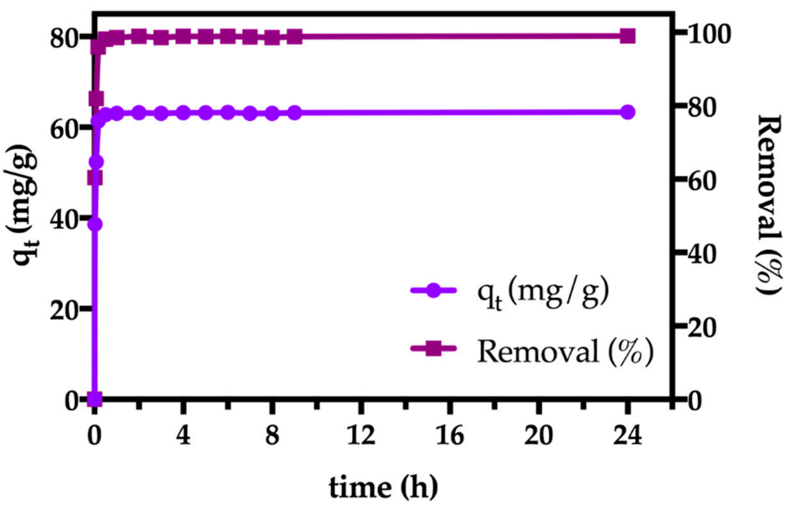

Figure S5. The effect of contact time on the removal of paraquat from water and adsorption capacity using the κ-carrageenan hybrid nanoparticles as sorbent.

C. Kinetic modelling

The adsorption kinetics is a fundamental parameter for the application of a material at industrial level. Several models have been established to facilitate the understanding of the adsorption kinetics and the rate-limiting step of a material in relation to an adsorbate. For the κ-carrageenan particles, three of the most commonly employed models were used to understand the kinetic profile of paraquat to these particles. The equations associated to each model are detailed below [53].

Pseudo 1st order

The Lagergren's first-order kinetics based on solid capacity is described as follows (S1)[50]:

$$\frac{dq}{dt} = k_1(q_e - q) \tag{S1}$$

The integration of the previous equation with the boundary conditions of t = 0, q = 0 and t = t, q = q results (S2):

$$\ln(q_e - q) = \ln q_e - k_1 t \tag{S2}$$

This equation can then be expressed as follows (S3):

$$q_t = q_e(1 - e^{-k_1 t}) \tag{S3}$$

where, q_t and q_e (mg.g^{-1}) are the adsorption capacity at time t and equilibrium time, respectively; k_1 is the equilibrium rate constant of pseudo 1st order adsorption (min^{-1}).

Generally, the pseudo-first order kinetics does not result in a good fit in the whole time interval and is usually applicable to the initial part of the kinetic profile.

Pseudo 2nd order

The pseudo 2nd order model relies on the sorption capacity of the solid phase, being expressed as follows (S4)[51]

$$\frac{dq}{dt} = k_2(q_e - q)^2 \tag{S4}$$

The integration of the previous equation with the boundary conditions of t = 0, q = 0 and at t = t, q = q, result in (S5):

$$\frac{1}{q_e - q} = \frac{1}{q_e} + k_2 t \quad (S5)$$

The previous equation can then be expressed as follows (S6):

$$q_t = \frac{k_2 {q_e}^2 t}{1 + k_2 q_e t} \quad (S6)$$

where, q_t and q_e (mg. g^{-1}) are the adsorption capacity at time t and equilibrium time, respectively; k_2 is the equilibrium rate constant of pseudo 2nd order adsorption (g.mg^{-1}.min^{-1}). Since the pseudo 2nd order rate constants can be determined experimentally, it provides a more appropriate representation of the kinetic data in adsorption systems.

Elovich

The Elovich fitting is usually applied when it is assumed that the solid surfaces are energetically heterogeneous[52]. The Elovich model is represented as follows (S7):

$$q_t = \frac{1}{\beta}\ln(\alpha\beta) + \frac{1}{\beta}\ln(t) \quad (S7)$$

where, α and β are the Elovich coefficients and define the initial sorption rate (mg. g^{-1}.min^{-1}) and the desorption constant respectively (g.mg^{-1}).

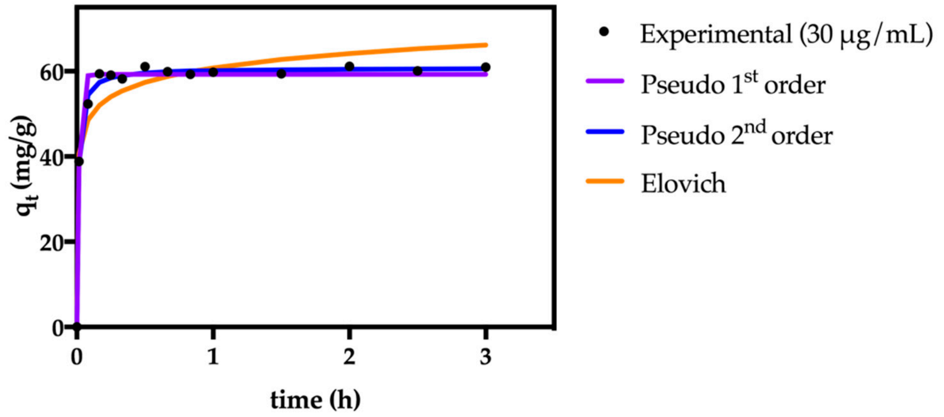

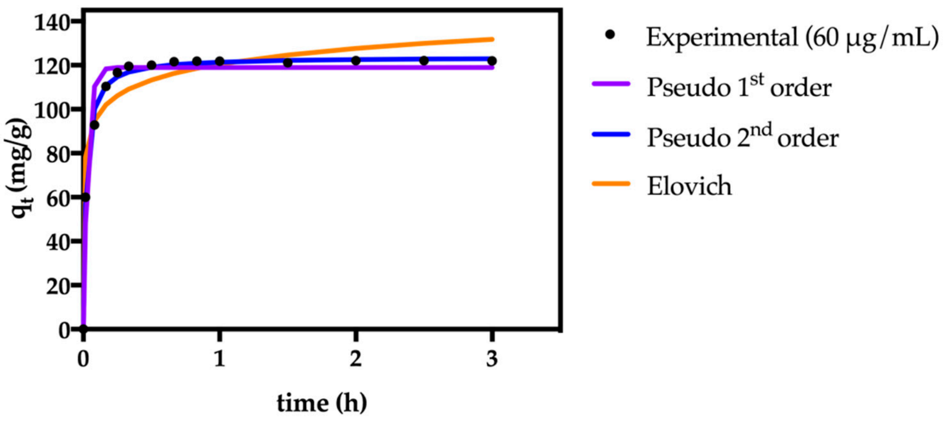

Figure S6. Modeling of adsorption kinetics of paraquat onto Fe_3O_4@SiO_2/SiCRG particles using pseudo 1st and 2nd order kinetic equations and Elovich kinetic model, for an initial paraquat concentration of 30 (top) and 60 µg/mL (bottom).

D. Equilibrium Isotherm Modelling

Several equilibrium isotherm models can be used to evaluate the efficacy of the adsorbent. There are many equations that can be used to describe the adsorption equilibrium of an adsorbate on an adsorbent. Under constant temperature conditions, the adsorption isotherms will describe the relationship between the amount of adsorbed adsorbate (q_e) and the adsorbate concentration in solution after the equilibrium is reached (C_e)[53,56,57]. In this work, the most common isotherm models were fit to the experimental data for a complete analysis of the k-carrageenan nanoparticles' performance. The adequacy of each model and the respective equation is presented below.

Langmuir isotherm

The Langmuir sorption isotherm applies to systems where the adsorption of the solute occurs as a monolayer on a surface containing a finite number of identical sites. The adsorption must be uniform with no transmigration of adsorbate in the surface plane[22,54,57,58]. The non-linear form of the Langmuir model is given by the following equation (S8):

$$q_e = \frac{q_L K_L C_e}{1 + K_L C_e} \tag{S8}$$

where, q_L (mg/g) is the monolayer adsorption capacity per unit of adsorbent and K_L (L/mg) is the Langmuir adsorption constant related to the affinity of binding sites.

Freundlich isotherm

The Freundlich isotherm assumes that the adsorption occurs on a heterogeneous surface, resulting in adsorption sites of varying energy. Since this model is based on an exponential equation, it assumes that the concentration of adsorbate on the adsorbent surface increases with the increase of adsorbate concentration. In general terms, this model assumes that the adsorption can occur based on multiple layers[22,54,57,59]. The non-linear form of the Langmuir models is described based on the following equation:

$$q_e = k_f {C_e}^{\frac{1}{n}} \tag{S9}$$

where k_F ($mg^{(1-1/n)}.L^{(1/n)}.g^{-1}$) is the Freundlich constant and 1/n is the heterogeneity factor which varies between 0 and 1.

Sips or Langmuir-Freundlich isotherm

The Sips model is based on the combination of the Langmuir and Freundlich isotherm models. At low adsorbate concentration, the Sips equation reduces to the Feundlich isotherm, however it predicts a monolayer adsorption at high concentration that is characteristic of the Langmuir isotherm[22,54,57,60]. This model takes the following non-linear form (S10):

$$q_e = \frac{Q_{max} K_s C_e^{\frac{1}{ns}}}{1 + \ K_S C_e^{\frac{1}{ns}}} \tag{S10}$$

where Ks is the Sips equilibrium constant ($(mg.L^{-1})^{-1/n}$ and Q_{max} is the Sips maximum adsorption capacity ($mg.g^{-1}$) and n_S is Sips model exponent. It is assumed that n_S should be between 0 and 1 for integration purposes.

Besides of the usual isotherm models, other fittings were also accomplished for the κ-carrageenan nanoparticles using alternative models. The equations regarding these fittings are detailed in the table below.

Table S1. Other isotherm models that were fit for adsorption of paraquat using the κ-carrageenan nanoparticles as adsorbent[22,54,57,61-63].

Model	Equation (non-linear form)	Parameters
Toth	$q_e = \frac{Q_{max} K_T C_e}{(1 + (K_T C_e)^{\frac{1}{n_T}})^n T}$	Q_{max} is the maximum sorption capacity ($mg.g^{-1}$)<br>K_T is the Toth equilibrium isotherm constant ($L.mg^{-1}$)<br>n_T is the Toth exponent (dimensionless)
Liu	$q_e = \frac{Q_{max}(K_g C_e)^{n_L}}{1 + (K_g C_e)^{n_L}}$	Q_{max} is the maximum sorption capacity ($mg.g^{-1}$)<br>K_g is the Liu equilibrium isotherm constant ($L.mg^{-1}$)<br>n_L is the Liu exponent (dimensionless) (> 0)
Hill	$q_e = \frac{Q_{max} C_e^{n_H}}{KH + C_e^{n_H}}$	Q_{max} is the maximum sorption capacity ($mg.g^{-1}$)<br>K_H is the Hill equilibrium isotherm constant $[(mg.L^{-1})^{nH}]$<br>n_H is the Hill exponent (dimensionless)

The goodness of the fittings was determined based on the calculation of the correlation coefficient (R^2) (S11) and Chi-square test value (χ^2) (S12), expressed by the following equation respectively:

$$R^2 = 1 - \frac{\sum_{i=1}^{n}(y_i - \hat{y}_i)^2}{\sum_{i=1}^{n}(y_i - \bar{y})^2} \quad \text{(S11)}$$

$$\chi^2 = \sum_{i=1}^{n} \frac{(y_i - \hat{y}_i)^2}{\hat{y}_i} \quad \text{(S12)}$$

where y_i and $\hat{y}_i$ are the experimental and model predicted values respectively, $\bar{y}$ is the mean of the experimental data and n is the sample size.

The standard free energy change (ΔG^o) was determined using the equation (S13) [66]

$$\Delta G° = -RT \ln K \quad \text{(S13)}$$

where K is the equilibrium constant for the sorption interaction, T is the temperature in Kelvin and R is the gas constant (8.314 J/(mol.K)). The value of K was calculated using the method suggested by Khan and Singh[65]. This method presupposes that at low concentration the sorption isotherm is a straight line, hence simulating a partitioning process and the equilibrium constant ($K_{KL}=q_e/C_e$) is calculated from the intercept on the ordinate of the plot $\ln(q_e/C_e)$ against q_e (see Figure S7).

The value of ΔG^o obtained for the adsorption of MTP on $Fe_3O_4@SiO_2$/SiCRG particles, at 25 °C was -25.19 kJ/mol.

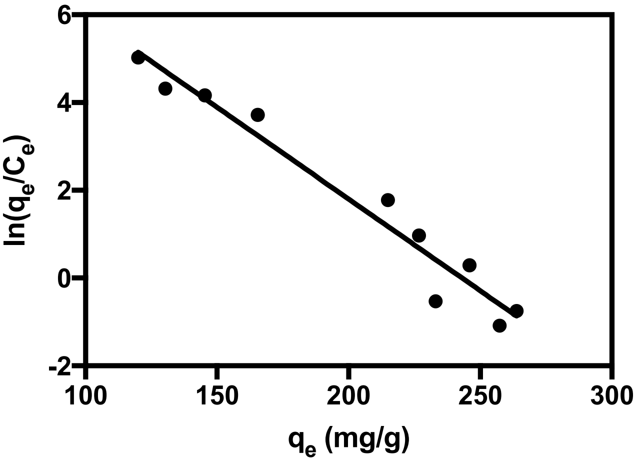

Figure S7. The method suggested by Khan and Singh for the calculation of K.

E. Quantification of Paraquat

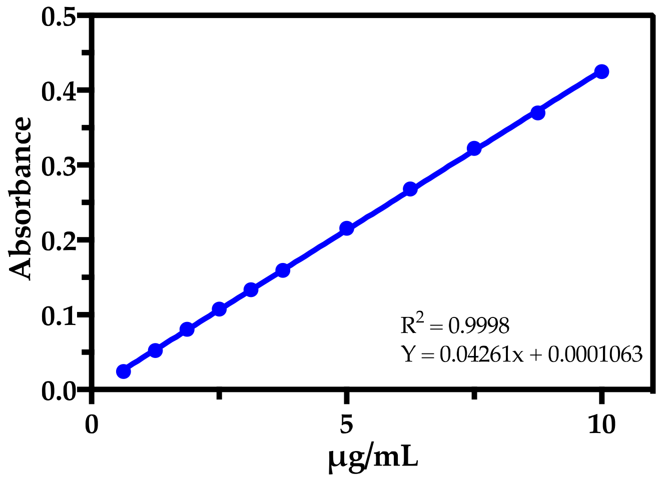

Figure S8. Calibration curve for the quantification of paraquat in water.
